# Is Legionellosis Present and Important in Colombia? An Analyses of Cases from 2009 to 2013

**DOI:** 10.7759/cureus.1123

**Published:** 2017-03-28

**Authors:** Andrés Mauricio Patiño-Barbosa, Andrés Felipe Gil-Restrepo, Valentina Restrepo-Montoya, Wilmer E. Villamil-Gomez, Jaime A. Cardona-Ospina, Alfonso J. Rodriguez-Morales

**Affiliations:** 1 Faculty of Health Sciences, Universidad Tecnológica De Pereira; 2 Infectious Diseases and Infection Control Research Group, Hospital Universitario de Sincelejo; 3 Public Health and Infection Research Group, Faculty of Health Sciences, Universidad Tecnológica De Pereira

**Keywords:** legionellosis, legionella, epidemiology, colombia, diagnosis, bacteria, respiratory infections

## Abstract

Infection due to *Legionella pneumophila *has been not studied in Colombia, although it is present. The observational, retrospective study in which the incidence of legionellosis in Colombia, 2009-2013, was estimated based on data extracted from the personal health records system (*Registro Individual de Prestación de Servicios*, RIPS) using the ICD-10 codes A48.1 (Legionnaires' disease) and A48.2 (Pontiac Fever). Using official population estimates of the National Administrative Department of Statistics (DANE), crude and adjusted incidence rates were estimated (cases / 100,000 pop). During the period, 206 cases were reported (mean of 41.2 per year) for the cumulated national rate of 0.45 cases / 100,000 pop. The clinical form of legionellosis with the highest incidence rates was the non-pneumonic Legionnaires' disease (0.39 cases / 100,000 pop) with women being the main affected (0.42 cases / 100,000 pop). The territory with the highest incidence rate was Bolivar department (1.94 cases / 100,000 pop), followed by La Guajira (1.7 cases / 100,000 pop). Finally, age groups with the highest morbidity were 0-9.999 years old (1.16 cases / 100,000 pop) and system of identification for social subsidies beneficiaries (SISBEN) category with the highest number of total cases was level one (88 cases). According to these results, we can show that legionellosis in Colombia is more common than it could be thought. Nevertheless, cross-sectional and prospective studies should be conducted in our country in order to improve the knowledge of incidence, prevalence, and burden of disease.

## Introduction

Legionellosis is an infectious disease caused by the gram-negative bacilli, *Legionella pneumophila* and other species belonging to the genus *Legionella*. It causes two types of conditions that vary in severity; a mild illness called Pontiac fever and the potentially fatal Legionnaires' disease. Both occur most frequently in elderly patients, people with immunosuppressive conditions as well with the history of a bronchial disease or exposed to various risk factors such as smoking [1-2].

The *Legionella *species are distributed worldwide in various aquatic environments both natural and man-made and can be acquired in the community, through hospital infections, or associated with travels through contaminated water sources.

Its usual form of presentation is in sporadic cases (the most common) and outbreaks; the latter is generally related to touristic (e.g. hotels) or hospital environments, both by contamination of water supply systems. Especially in this setting, diagnostic considerations are very important. While pneumonia caused by numerous pathogens share similar laboratory findings, hyponatremia (sodium < 130 mEq/L) secondary to the syndrome of inappropriate antidiuretic hormone (SIADH) is more common in Legionnaires' disease than in pneumonia secondary to other pathogens; however, this is not specific for Legionnaires' disease. Nevertheless, the definitive method for diagnosing *Legionella* is the isolation of the organism in the respiratory secretions (i.e. sputum, lung fluid, pleural fluid). The most widely used are serological tests which include the indirect fluorescent antibody (IFA) and enzyme-linked immunosorbent assay (ELISA) tests. A single increased antibody titer confirms Lyme disease (LD) if the IFA titer is greater than or equal to 1:256.

From the total number of exposed cases, about 95% develop Pontiac fever and only 0.5 to five percent develop the Legionnaires' disease, whose lethality reaches up to 15%.

Epidemiological studies have been made in countries such as Spain showing the incidence, prevalence, and mortality of the disease, besides other reports of outbreaks of Legionnaires' disease in this country and in the United States of America and Europe [3-6]. In Colombia, although there are a few studies, mostly case reports, addressing this condition, none have made estimations of morbidity caused by this bacterial disease.

Based on the above, using a Health Information System called the Individual Records of Health Service (*Registro Individual de Prestacion de Servicios, *RIPS), from the Colombian Ministry of Health [7], we have proceeded to retrieve diagnosed cases and estimate the incidence of legionellosis in Colombia.

## Materials and methods

For this observational, retrospective study, the epidemiological data was collected from the so-called personal health records system (Registro Individual de Prestacion de Servicios, RIPS). The International Classification of Diseases 10th version (ICD-10) codes A48.1 and A48.2 were used, given the fact that legionellosis is not under the surveillance system, to obtain the number of cases from each department of the country by year (2009-2013). Data was obtained with agreement from the Ministry of Health through the Protection Information System Sistema Integral de Información de la Protección Social Ministerio de Salud y Protecci (SISPRO) through a client access server which allowed retrieving cases from the SISPRO server to a local computer. SISPRO RIPS data used for this study are constituted from confirmed cases, which have been revised in terms of data quality, initially from data from Colombia and later by SISPRO and its Data Cubes system. Data proceeded for this study from 33 reference notification units, one per department, and was later consolidated and centralized in Bogota up to the SISPRO system. Currently revised and consolidated data is available for the period 2009-2013. Finally, the quality of RIPS data in Colombia has been described elsewhere [7].

Using official reference population data (National Administrative Department of Statistics, DANE), estimates of annual incidence rates for all departments of the country during the study period were calculated (32 departments and the capital district, for five years) (cases/100,000 pop) to provide, for the first time, estimates of the legionellosis incidence in the country by department. Incidence rates were estimated by age-group.

During the study period, the diagnostic methods used were the same. In the case of the serological tests, the reported sensitivity of such tests was >95%.

## Results

During the period, 206 cases were diagnosed and reported (average of 41.2 cases per year), for a cumulated national rate of 0.45 cases / 100,000 pop. The clinical form of legionellosis with the highest incidence rates was the non-pneumonic Legionnaires' disease with 0.39 cases / 100,000 pop (183 cases), being female, the sex more affected with 0.42 cases / 100,000 pop (95 cases).

**Figure 1:**
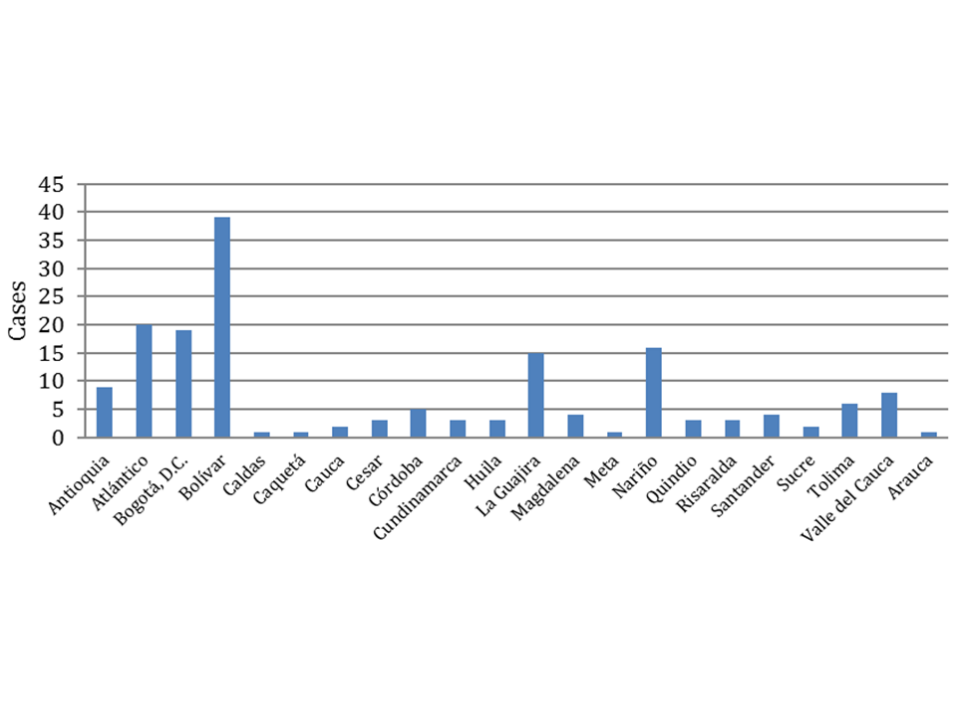
Non-pneumonic legionellosis, Colombia, by departments

The department with the highest incidence rate of non-pneumonic Legionnaires' disease was Bolivar in the north of the country (1.94 cases / 100,000 pop), followed by La Guajira also in the north (1.7 cases / 100,000 pop); while the department with the lowest incidence rate was Caldas, in the Andean area (0.1 cases / 100,000pop). The highest incidence rate of pneumonia Legionnaires' disease was Meta (0.67 cases / 100,000 pop), followed by Bogota, D.C., the capital (0.05 cases / 100,000 pop) (Figure 1).

According to location, 57% of cases were in the urban area, pneumonic Legionnaires' diseases 39% vs 59.56% of non-pneumonic Legionnaires' disease and 14.07% in the rural zone. In 30.09%, information about localization was not available.

**Figure 2:**
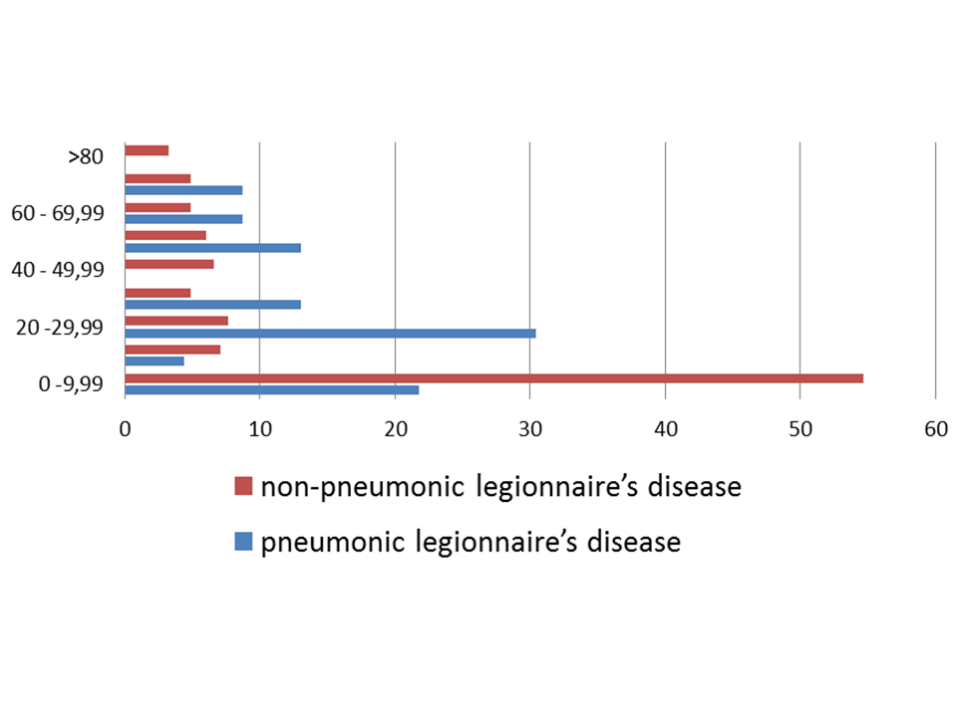
Age distribution (%) of cases of legionellosis, Colombia, 2009-2013

Finally, by age groups, those with the highest morbidity were 0-9.999 years-old (1.16 cases / 100,000 pop) in non-pneumonic Legionnaires' disease, while pneumonic Legionnaires' diseases were 70-79,999 years-old (0.13 cases / 100,000 pop). According to the national social classification, most cases belong to the low and vulnerable level, the System for the Selection of Beneficiaries for Social Programs (SISBEN) category one, 88 cases (Figure 2).

## Discussion

According to our results, the incidence of Legionella infection is not only present in the country, but also has been increasing since 2009 until 2013, with a peak of cases in 2012 (76 cases) and a cumulative incidence of 0.45 cases per 100,000 inhabitants, being the non-pneumonic infection form, with 183 cases, the most common form of the disease presentation, which is a finding similar to those reported in the literature.

Our findings show that the incidence was higher in the departments of the north Caribbean coast of the country, Bolivar and La Guajira. Legionellosis has been reported in other Latin American countries such as Chile [8-9], Venezuela [10], Argentina [11] Mexico [12], Brazil [13-14] and Cuba [15], nevertheless the research and publication on legionellosis is still very limited, particularly in Latin America.

Of the 206 cases reported here, from Colombia, 118 occurred in municipal urban areas, which could be consistent with data found in the city of Sao Paulo, Brazil, where in 2007 *Legionella *infections were detected in urban areas of the metropolitan city [16]. In addition, not all cases were found in areas with high population density in 2012 in Colombia. A study in Colombia showed the presence of *Legionella *in a sample of surface waters in the Los Nevados National Natural Park, a rural mountain area [17]. In 2015 in Costa Rica and Nicaragua, *Legionella *ssp. were isolated from banana plantations [18].

It is noteworthy that a large number of cases occurred in patients with the lowest socioeconomic classification (SISBEN category one) (88 cases), with an increase in a number of cases until 2012 and a substantial reduction in 2013. With regard to age groups, the highest number of cases occurred in the zero to nine-year-old group, with 105 cases, contrasting with the literature, which said the infection is most prevalent in people over 50 years old [19-20]. This would be related to multiple factors, including sub-record of cases in the older population, where usually respiratory tract infection does not include *Legionella *ssp. as a differential diagnosis [21]. Our findings indicate that the male: female ratio is 1:08, 1 for non-pneumonic Legionnaires' disease, which is consistent with other reports in which legionella infection is more prevalent in men [20].

Finally, although the study presents multiple limitations, particularly because it is based on secondary information, we can say that its results on *Legionella *infection in Colombia show that this pathogen is more common than thought. Then, more studies about it are necessary to better focus the epidemiology of the disease as well its clinical implications.

## Conclusions

According to these results, we can say that legionellosis is present in Colombia and is more common than it could be thought. Nevertheless, cross-sectional and prospective studies should be conducted in our country in order to improve the knowledge of incidence, prevalence, and burden of the disease.
